# Quantifying acceptable artefact ranges for dermatologic classification algorithms

**DOI:** 10.1002/ski2.19

**Published:** 2021-03-19

**Authors:** T.C. Petrie, C. Larson, M. Heath, R. Samatham, A. Davis, E.G. Berry, S.A. Leachman

**Affiliations:** ^1^ Department of Dermatology Oregon Health & Science University Portland Oregon USA

## Abstract

**Background:**

Many classifiers have been developed that can distinguish different types of skin lesions (e.g., benign nevi, melanoma) with varying degrees of success.^1–5^ However, even successfully trained classifiers may perform poorly on images that include artefacts. While problems created by hair and ink markings have been published, quantitative measurements of blur, colour and lighting variations on classification accuracy has not yet been reported to our knowledge.

**Objectives:**

We created a system that measures the impact of various artefacts on machine learning accuracy. Our objectives were to (1) quantitatively identify the most egregious artefacts and (2) demonstrate how to assess a classification algorithm's accuracy when input images include artefacts.

**Methods:**

We injected artefacts into dermatologic images using techniques that could be controlled with a single variable. This allows us to quantitatively evaluate the impact on the accuracy. We trained two convolutional neural networks on two different binary classification tasks and measured the impact on dermoscopy images over a range of parameter values. The area under the curve and specificity‐at‐a‐given‐sensitivity values were measured for each artefact induced at each parameter.

**Results:**

General blur had the strongest negative effect on the *melanoma* versus *other* task. Conversely, shifting the hue towards blue had a more pronounced effect on the *suspicious* versus *follow* task.

**Conclusions:**

Classifiers should either mitigate artefacts or detect them. Images should be excluded from diagnosis/recommendation when artefacts are present in amounts outside the machine perceived quality range. Failure to do so will reduce accuracy and impede approval from regulatory agencies.

## Graphical abstract

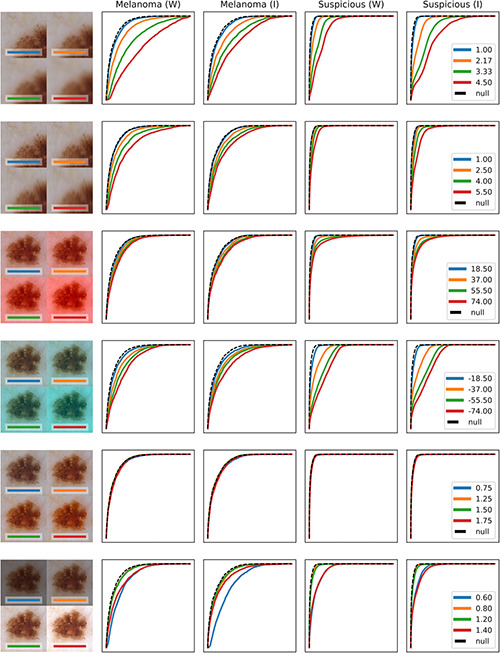

Machine learning classification relies on various measures of accuracy but with dermatologic images, these are substantially dependent on the presence and degree of artefacts. We propose including machine perceived image quality assessments to define acceptable ranges of artefacts prior to attempting to classify images. We demonstrate such an approach using images from the ISIC Archive.

1

**What is already known about this topic?**

- Image artefacts reduce diagnostic accuracy for both practitioners and machine learning algorithms. Visual specialities such as ophthalmology and radiology are actively engaged in Image Quality Assessment (IQA). IQA seeks to quantify ‘human perceived image quality’. In dermatology, image quality is acknowledged as an issue but is typically reported in subjective terms. With few exceptions, the impact of artefacts on machine learning of dermatologic images has not been studied.

**What does this study add?**

- Operational qualification testing is required for clinical laboratory devices. So too, machine learning algorithms should be able to identify which images are outside their range of interpretation. We introduce the idea of ‘*machine* perceived image quality’ to quantitatively report the impact of different artefact classes on machine learning algorithms. Artefacts are modelled on real‐world MoleMapper™ data. This study demonstrates a method for establishing such ranges for artefact classes.

## 2 INTRODUCTION

For at least 3 decades, attempts have been made to diagnose melanoma from digital images using computers.^6^ This effort has been relatively continuous over that period and recent advances both in the proliferation of smartphones and the successes of machine learning have only accelerated the research in this area. The dominant machine learning approach to lesion diagnosis (clinical term) or classification (computer science term) of images is to *train* a convolutional neural network (CNN). This training is usually performed in a supervised fashion by providing images with labels (e.g., diagnoses) which the network learns iteratively. Trained networks can then be used to perform *inference* on previously unseen images assigning the most probable label (diagnosis) to the new image. Historically, research on this topic has been prematurely interpreted as ready for translation into either clinical or personal‐use tools (e.g., the proposed decision support system trained on 358 images^7^ or the Internet based screening system trained on 1258 images^8^). This is especially problematic for smartphone apps.^9^, ^10^, ^11^

Nevertheless, the objective of building tools to augment clinical diagnostic efforts or to triage lesions for people who live in underserved regions is important. False starts are not a sufficient reason to abandon efforts, but all ongoing efforts need to proceed with proper caution and evidence‐based approaches. In this paper, we demonstrate such an approach with respect to image artefacts, a common and formidable obstacle in dermatological images.

Artefacts have become problematic for datasets used in dermatology machine learning tasks because the data often comes from a wide variety of undocumented camera and dermoscopy equipment whose capabilities have changed rapidly over the large timespan during which the images were collected. These data exhibit colour variations, saturation variations and intensity variations. They also exhibit different kinds of blur and inclusion artefacts such as bubbles, hair, rulers and black corners due to dermatoscope field of view limitations.

These artefacts also pose challenges for dermatologists providing telehealth services. Poor‐quality images make consultation or diagnosis difficult and risky. The ability to detect bad images prior to submission into a telehealth system could reduce the time spent by providers rejecting images and by patients waiting for answers.

The goal of this research was not to produce a top‐of‐the‐leader‐board classifier but rather to quantitatively study the impact of artefacts specifically on machine learning classification. To do that, we needed to train a classifier and measure how its performance degraded on images with increasingly severe artefacts. Since most image classifiers now use CNNs in some fashion, we chose this as our general classifier architecture. To avoid simply learning how artefacts behave with a specific CNN architecture, we trained two different CNNs that have been reported in the literature. To further generalize our results, we also trained each classifier on two different classification tasks (the diagnosis and the recommendation tasks). We use established metrics commonly used in the field.

## 3 MATERIALS AND METHODS

### 3.1 Dataset

The International Skin Imaging Collaboration (ISIC) archive^12^ is a publicly available repository of over 23 000 digitized dermoscopy images with clinical labels that has been widely used in image analysis research on skin lesions.^13^ ^,^ ^14^ The dataset includes several thousand nevus images that contain large coloured marking disks near the lesion. Early experiments showed that our CNNs were using those disks as cues and learning on them. We also noted many images contained black corners, rulers, ink markings and other inclusions. Many times, the lesion of interest was a very small portion of the image and the resizing needed by the CNNs caused small features to be lost. The Inception‐v3 CNN required the images to be square but the ISIC images are rectangular and the resulting reshaping created new resizing artefacts we did not intend to study. For those reasons, we chose to manually crop the images and train/test on the cropped images.

Our cropping rule was to keep the cropping region roughly square and retain a margin around the lesion no greater than the lesion's diameter. Where possible, we cropped out the various inclusions.

### 3.2 Classification tasks

We used images from the ISIC Archive which are labelled. These labels include ‘diagnosis’ and ‘diagnostic method’ attributes. Using these attributes, we partitioned the data into two different binary datasets.

We defined the first binary *diagnosis classification* task as *melanoma* versus *other* where ‘other’ included the following diagnosis labels: actinic keratosis, angioma, atypical melanocytic proliferation, basal cell carcinoma, dermatofibroma, lentigo NOS, lentigo simplex, nevus, other, pigmented benign keratosis, seborrheic keratosis, solar lentigo, squamous cell carcinoma and vascular lesion.

For the *management decision* task recommending *biopsy or follow*, dermatologists in our group helped create a general set of rules to relabel the images. To generate the decision labels from the ISIC labels, we used the following criteria.

- Anything with a diagnosis of actinic keratosis, atypical melanocytic proliferation, basal cell carcinoma, melanoma or squamous cell carcinoma was marked ‘biopsy’ regardless of diagnostic method.
- Remaining data with a diagnostic method of ‘histopathology’ were included in the ‘biopsy’ set. Even if the diagnosis returned ‘nevus’ we considered it suspicious if a clinician was in enough doubt biopsy it.
- Data with no diagnostic method specified were excluded from the dataset.
- Data with a diagnostic method specified (but not ‘histopathology’) and had a diagnosis of angioma, dermatofibroma, lentigo NOS, lentigo simplex, nevus, seborrheic keratosis, pigmented benign keratosis or solar lentigo were labelled ‘follow’.
- Any remaining data not fitting these criteria were excluded from the dataset.

In both partitions, we dropped images that were either missing a diagnosis or missing a diagnostic method leaving 23 135 samples in the management decision task and 23 646 samples for the diagnosis classification task.

### 3.3 Data preparation

The relabelled datasets described above were split into testing and training data with an 80/20 split into the training/testing sets. Each set was further designed such that 40% of the data was labelled with the positive class (‘melanoma’ or ‘biopsy’ depending on the task) and 60% was the negative class (‘other’ or ‘follow’). To minimize class bias errors, class imbalance was compensated for by using class weights on the loss function such that the smaller class errors carried more weight. We performed Monte Carlo Cross Validation splitting the data randomly (bootstrap with replacement) on each training/testing iteration. Results from nine iterations were averaged together for the final results.

In the case of the *diagnosis* task, the constraining factor was the total number of available melanoma samples. Preserving the training/testing and class ratios resulted in 4319 images in the training set of which 1728 were labelled melanoma. In the case of the *management decision* task, there were many more ‘biopsy’ samples resulting in 17 729 images in the training set of which 7092 were labelled biopsy.

To compensate for small numbers in the datasets, data were augmented by resizing images down to 350 × 350 and randomly cropping a 299 × 299 image from those. The 299 size was chosen because of *Inception‐v3* requirements and kept for the *Wide ResNet* model for comparative consistency. Once cropped, images were randomly flipped horizontally and vertically using a standard 50–50 Bernoulli trial. This allowed us to substantially increase our effective datasize.

Pretrained PyTorch models expect images to be colour normalized so for both fine‐tuned transfer learning and inference, the ISIC image colour channels were normalized as per the PyTorch documentation during the image loading process.

### 3.4 Framework and training

We implemented our system using the PyTorch 1.2 framework on Python 3.7 using CUDA 10.1 libraries to take advantage of an NVIDIA GeForce GTX 1060 GPU running on a Linux (Ubuntu 16.04) system with 16‐GB RAM and an Intel Core i7‐6700K CPU for training and inference.

We used the *Inception‐v3* and *Wide ResNet‐101‐2* models^15^ ^,^ ^16^ pretrained on the ImageNet dataset^17^ ^,^ ^18^. The *Inception‐v3* model was used in the seminal paper by Esteva et al.^5^ while the *Wide ResNet* model is a more recent CNN. These models were then fine‐tuned using transfer learning with the new labels.^19^ This was done by replacing the final fully connected 1000‐class layer with a single fully connected binary layer (with random weights). Training was done using stochastic gradient descent optimizer with a learning rate of 0.01, momentum of 0.5 and batch sizes of 8 to stay within memory constraints on the GPU. Backpropagation was allowed to change all the weights in the network. The loss function for these binary classification networks was the typical binary cross‐entropy with sigmoid loss implemented with PyTorch's BCEWithLogitsLoss function. The relatively small dataset sizes described in detail below resulted in the networks quickly overfitting while using the default built‐in regularization in the networks. Training for 12 epochs was empirically found to be a reasonable compromise by examining learning curves constructed from training and validation set performance. This yielded networks trained to recognize the diagnostic and recommendation classes we defined for the dermoscopy images.

### 3.5 Artefact creation and testing

We synthetically generated images with artefacts using a single numerical parameter to control the amount of artefact introduced into each test image. Based on observations from the MoleMapper™ data, we studied the following classes of artefacts.

#### 3.5.1 Blur

We simulated image blur by convolving the images with a Gaussian kernel whose single parameter σ is used for both *x* and *y* directions and represents the standard deviation of the normal curve measured in pixels. This is similar to the work of Vasconcelos and Rosado.^20^ This simulates image blurring caused by the object of focus being outside of the depth of field or by motion of the camera along the optical axis.

#### 3.5.2 Motion

We simulated artefacts created when the user moves the camera (e.g., slight hand shaking) parallel to the surface being photographed using a Gaussian kernel but only in one direction.

#### 3.5.3 Red colour shifts

To simulate the red shift observed in images, we measured the average of the standard deviation of the dataset for the red channel and increased the red values in the test images by multiples of the standard deviation. To prevent saturation, we set the maximum possible value of the mean to one standard deviation below the highest possible value (255).

#### 3.5.4 Blue colour shifts

To simulate the blue shift observed in images, we found that simply lowering the values of the red channel was enough to mimic this visually. Using the same red channel standard deviation value for the red‐shift case, we subtracted multiples from the red channel in the test images. To prevent under saturation, we set the minimum possible value of the mean to two standard deviations above the lowest value (0).

#### 3.5.5 Saturation

Differences in saturation tend to come from different models of smartphones. We mimicked observed saturation differences using the PyTorch adjust_saturation function in the transforms.functional module.

#### 3.5.6 Intensity

Differences in intensity can also be seen with different models of smartphones as well as lighting variation. We mimicked observed intensity differences using the PyTorch adjust_brightness function in the transforms.functional module.

## 4 RESULTS

**FIGURE 1:**
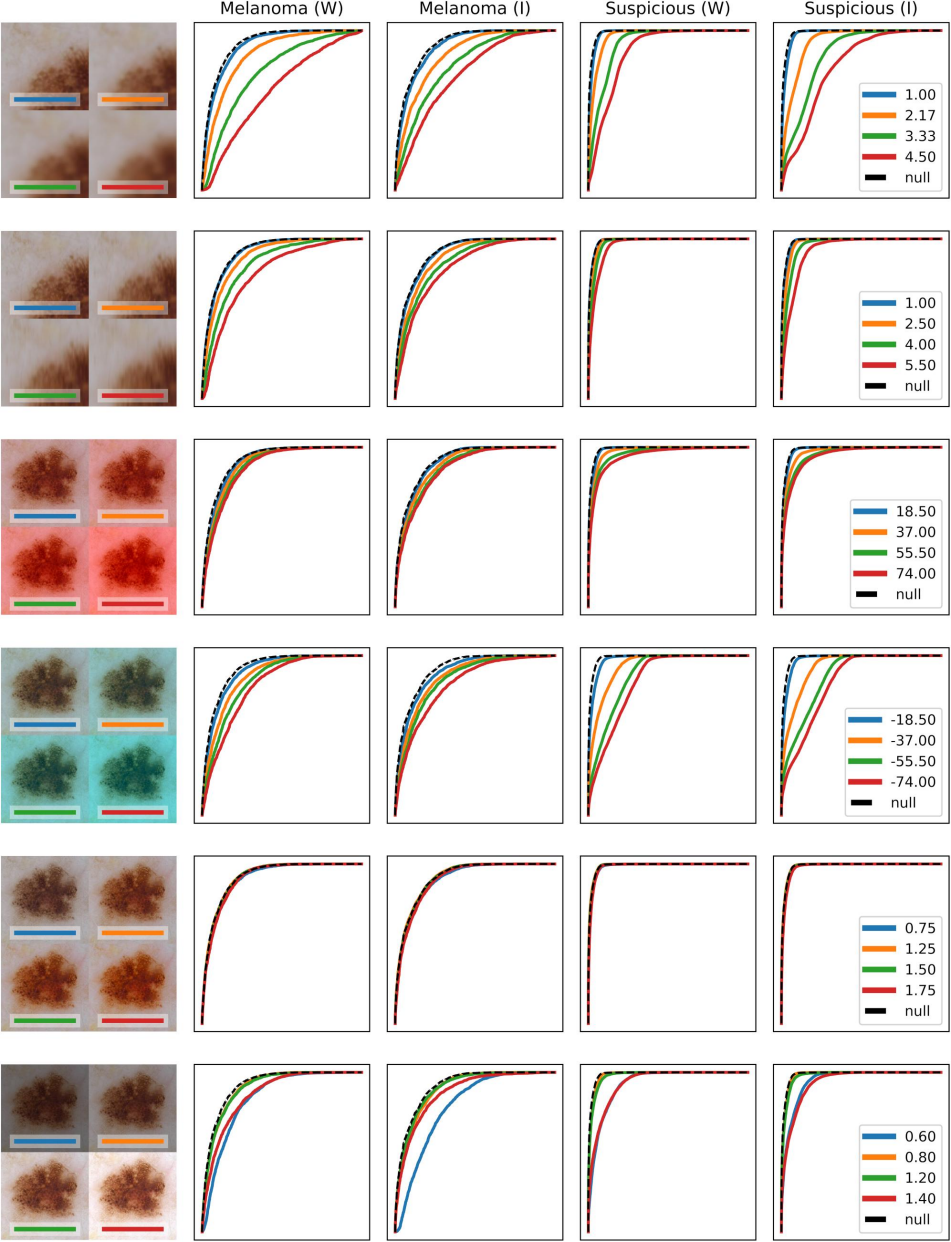
Each row represents the effect of injecting a particular artefact. The composite image at the left shows the effect of adding the artefact at test levels to an example image. ROC curves show sensitivity (*y* axis) against the false positive rate (*x* axis). Legends show the parameter values under test. ROC columns compare *melanoma* versus *other* and *biopsy* versus *follow* tasks for the Wide ResNet (W) and Inception (I) CNNs. CNN, convolutional neural network; ROC, receiver operating characteristic

We report the results in Figure 1 using the generally reported area under the curve (AUC) on receiver operating characteristic (ROC) curves. To assess algorithm performance in terms of clinical performance, we also report specificity values at defined sensitivity operating points. This method is also used by Marchetti et al.^13^ and repeated by Codella et al.^14^ who performed a similar analysis for their classification results.

Table 1 shows the impact of artefacts compared against the control case of no artefact. The process is repeated for images distorted by each class of artefact. The sensitivity control points were chosen using average sensitivity values of dermatologists on similar diagnosis and management tasks reported in Table 1 in Marchetti et al.^13^ We used values from Table 1 in Haenssle et al.^3^ who also report on similar tasks but differentiate between dermatologist skill level to calculate differences between average and expert dermatologists. We then treated our results from the control tests as expert practitioners. The differences between expert and average dermatologists were subtracted from the control specificity and AUC values to create ‘below average’ thresholds. Artefact tests resulting in values that dropped below these thresholds represent ‘below average’ performance and are shown in bold.

**TABLE 1:** Results of artefacts on AUC and specificity

		Diagnostic task				Management task			
		Wide ResNet		Inception v3		Wide ResNet		Inception v3	
	Parameter	AUC	Spec.	AUC	Spec.	AUC	Spec.	AUC	Spec.
Blur	1.00	0.91	0.83	0.90	0.81	0.98	0.95	0.98	0.95
	2.17	**0.85**	**0.74**	**0.85**	**0.71**	**0.96**	**0.91**	**0.93**	**0.85**
	3.33	**0.74**	**0.54**	**0.78**	**0.60**	**0.92**	**0.83**	**0.85**	**0.71**
	4.50	**0.62**	**0.35**	**0.72**	**0.49**	**0.87**	**0.75**	**0.78**	**0.56**
Motion	1.00	0.92	0.84	0.91	0.82	0.99	0.96	0.99	0.96
	2.50	0.89	**0.80**	0.88	**0.78**	0.98	0.95	0.98	0.94
	4.00	**0.84**	**0.72**	**0.85**	**0.73**	0.97	0.93	**0.96**	**0.88**
	5.50	**0.78**	**0.63**	**0.82**	**0.67**	**0.96**	**0.90**	**0.95**	**0.86**
More red	18.5	0.91	0.84	0.91	0.82	0.98	0.96	0.98	0.95
	37.0	0.90	0.81	0.89	0.79	**0.97**	0.93	**0.97**	0.92
	55.5	0.88	**0.79**	**0.88**	**0.76**	**0.96**	**0.90**	**0.96**	**0.88**
	74.0	**0.87**	**0.77**	**0.87**	**0.74**	**0.95**	**0.87**	**0.95**	**0.86**
More blue	−18.5	0.90	0.82	0.90	0.81	0.98	0.94	0.98	0.94
	−37.0	**0.87**	**0.77**	**0.87**	**0.76**	**0.94**	**0.83**	**0.93**	**0.82**
	−55.5	**0.85**	**0.72**	**0.85**	**0.73**	**0.88**	**0.73**	**0.87**	**0.71**
	−74.0	**0.81**	**0.67**	**0.82**	**0.66**	**0.84**	**0.68**	**0.84**	**0.66**
Brightness	0.60	**0.84**	**0.72**	**0.79**	**0.64**	**0.93**	**0.82**	**0.95**	**0.87**
	0.80	0.91	0.82	0.90	0.80	0.98	0.95	0.98	0.95
	1.20	0.90	0.82	0.90	0.82	0.98	0.94	0.98	0.95
	1.40	**0.86**	**0.74**	**0.87**	**0.76**	**0.93**	**0.82**	**0.94**	**0.84**
Saturation	0.75	0.91	0.84	0.90	0.82	0.99	0.96	0.98	0.96
	1.25	0.92	0.85	0.91	0.83	0.99	0.97	0.99	0.96
	1.50	0.92	0.84	0.91	0.82	0.99	0.96	0.98	0.96
	1.75	0.91	0.83	0.90	0.81	0.98	0.96	0.98	0.95
Control	0.0	0.92	0.85	0.91	0.83	0.99	0.97	0.99	0.96

*Note*: Columns contain the results from two CNNs for each task. Specificity is calculated at sensitivity = 0.84 for the diagnostic task and 0.89 for the management task. Values in bold represent a difference greater than between an Expert and Average dermatologist.

Abbreviations: CNN, convolutional neural network; AUC, area under the curve.

Looking at the impact of blur in Table 1, the *Wide ResNet* model was more robust on the management task, whereas the *Inception‐v3* model was more robust on the diagnostic task. Observe that *blur* is the most damaging artefact followed by colour shifts in the blue direction. ROC curves show that *motion blur* did not have as significant an impact possibly because information is lost mainly in one direction and there appears to be enough information in the remaining directions for the classifier to operate. This is important because it implies that CNNs are robust to some amount of human shake during image acquisition. *Brightness* was a problem at each extreme. *Saturation* seemed to have the least impact of any of the artefacts and, coupled with the impact of blur, indicates that fine features are important for classification. Given the relatively small size of the dataset, we consider the actual numbers to be preliminary but the process to be reasonable.

## 5 DISCUSSION

These results demonstrate the need to calibrate the range of acceptable artefacts on any given machine learning task. We have introduced the phrase ‘machine perceived image quality’ borrowing from the work on Image Quality Assessment research to describe this quantification effort. This effort underscores the concept that the performance of a classifier will vary widely across images with different impediments and that simple metrics of accuracy are insufficient to describe the usefulness of a classifier.

To arrive at the various thresholds used, we followed existing published work. In Marchetti et al.’s^13^ paper, sensitivity numbers were chosen by asking eight dermatologists to provide diagnosis on a *melanoma* versus *benign* diagnosis task and management decision on a *biopsy* versus *monitor/reassure* recommendation task and then averaging the results. These are the tasks we have emulated in our research. However, our diagnostic task (melanoma vs. other) is more difficult and hence we would expect both sensitivity and specificity to be lower. For the purposes of this work, we are only concerned with relative differences and so we deemed the tasks similar enough to use as a baseline.

We did not include bubble artefacts seen in dermoscopy images because the modality under study in our group, smartphone images, do not contain bubbles. We also did not include hair artefacts because they are difficult to simulate realistically and there is an existing body of work that is already focused on mitigating the effects.^21^, ^22^, ^23^, ^24^ Likewise, we did not include tattoo marks because of the difficulty in simulation but we would expect even worse outcomes than clinical ink markings.^25^

We have demonstrated a method for quantitatively evaluating the impact of six classes of artefacts on different tasks implemented using different CNN architectures. Our parameter choices were based on expert judgement after empirically studying our MoleMapper™ data. However, while we have shown that blurriness simulated with Gaussian kernels can have a strong negative impact on inference, we do not *quantitatively* know how closely our chosen values reflect the real‐world data. In follow‐up research, we intend to quantify the range of realistic simulation parameters evident in that data using validated computer vision techniques.

To fully understand the impact of these artefacts, we need to combine their effects, their prevalence and their parametric distribution. This is similar to calculating risk: determining the cost of an event times its probability. Our ongoing work to measure the prevalence of artefacts in over 8000 smartphone images of moles from our data, coupled with artefact impacts, will show us which artefacts to prioritize for detection and mitigation. For these reasons, it is important to continue to take an evidence‐based approach to understanding the impact of these artefacts so that the research community can prioritize their mitigation efforts on the critical classes of artefacts.

Ultimately it is the clinical community who will approve and recommend the use of devices that attempt to diagnose or triage skin conditions. Such devices are expected to be used by people without clinical training in a wide variety of settings. This will exacerbate the prevalence of artefacts thus increasing the number of poor‐quality images that generate questionable classifications which lead to erroneous recommendations. Failure by these devices to establish quantified ranges of acceptable artefacts, and refusing to make recommendations on images outside of these ranges, could reduce their accuracy in dangerous ways and preclude approval from regulatory agencies. The work presented here is intended to be another technique for evaluating such devices both internally and externally and to act as a foundation for minimizing out‐of‐range image classification.

## CONFLICT OF INTEREST

The authors declare that there are no conflict of interests.
